# Improving characterisation of human Multipotent Stromal Cells cultured in 2D and 3D: Design and evaluation of primer sets for accurate gene expression normalisation

**DOI:** 10.1371/journal.pone.0209772

**Published:** 2018-12-31

**Authors:** Bas Brinkhof, Huidong Jia, Bo Zhang, Zhanfeng Cui, Hua Ye, Hui Wang

**Affiliations:** Department of Engineering Science, University of Oxford, Oxford, United Kingdom; University Hospital Modena and Reggio Emilia, ITALY

## Abstract

Human Multipotent Stromal Cells (MSCs) are a valuable resource for regenerative medicine and are widely studied. They can be isolated from a variety of tissues and differentiate into multiple cell types (multi-potent). Many reports have been published using human MSCs and to be able to compare outcome, or be able to identify differences between MSCs, several cell surface markers have been proposed. Nevertheless, still many differences remain. Gene expression is known to be different between cell stage and origin. Furthermore, cells cultured on a culture dish (2D) show different gene expression profiles as compared to cells grown on scaffolds (3D). Even the RNA extraction method and the selection of genes used for normalisation have a role in gene expression profiling. To be able to compare gene expression data from samples cultured in different dimensions and RNA extracted using a variety of protocols we set out to define a set of reference genes suitable to normalise qPCR data from a very heterogeneous sample set. Hereto, Trizol was used to extract RNA from human MSCs cultured in 3D and 2D to validate newly designed and previously published primer sets. Subsequently, RNA from fresh human MSC samples and samples stored in RLT-buffer, Trizol or RNAlater was extracted using RNeasy and Trizol methods. All samples have been used to rank the candidate reference genes according to their stability after qPCR enabling identification of the most suitable reference gene(s) for normalisation of a heterogeneous sample set. The most stably expressed reference genes indicated superior normalisation of MSC marker gene expression over the least stable reference genes.

## Introduction

Multipotent stromal cells, also known as mesenchymal stem cells, mesenchymal stromal cells or medicinal signalling cells [1] (MSCs), hold great promise for regenerative medicine and tissue engineering. MSCs can be isolated from virtually all post-natal organs and tissues in the human body [2], are plastic adherent whilst maintaining multipotency when expanded, can differentiate *in vitro* into several distinctive end-stage cell types and possess immunoregulatory and regenerative capacity through secretion of bioactive macromolecules [3]. Because of their relatively easy isolation from many tissues the identity of the true MSC is unknown. The definition of the MSC phenotype is focussed on the expression of cellular surface markers (CD105, CD90 and CD73) or the lack thereof (e.g. CD45, CD34, CD19 and CD11b) [4]. These minimal requirements resulted in numerous reports using MSCs with variable outcome regarding maintaining multipotency or differentiation potency and efficiency. Other reasons why differences between MSCs can be observed are the age of the donor [5] and the duration of culture of the MSCs [6].

To properly perform MSC batch analysis, gene expression profiling could be utilised and quantitative PCR (qPCR) is the gold standard. Low amounts of starting material are required for qPCR and it has a very wide dynamic range. This technique is very useful if the target gene is known. If it is unknown which gene(s) will be differentially expressed, microarray might be the preferred method, though its dynamic range is very low. An alternative to microarray is RNA sequencing (RNAseq). Although a bit more expensive than microarray, the much broader dynamic range, possible discovery of new genes or splice variants and the ability to examine DNA variations are only some of the benefits [7]. These techniques require the extraction of RNA from MSCs cultured in 2D (on culture dish in monolayer) or 3D (cultured in a scaffold of any particular material). Several methods have been developed to extract RNA from cells and are available through even more suppliers. Usually, at least 100ng good quality RNA is required per sample to perform RNAseq. For lower amounts or significantly degraded RNA some solutions are available like RNA amplification kits or kits developed for low quality RNA, respectively. Nevertheless, as these kits can introduce biases, their use should ideally be avoided [8]. Microarray or RNAseq results should always be confirmed by qPCR and therefore there needs to be additional RNA remaining from the samples that can be used for microarray or RNAseq. In general, 1μg RNA is used to obtain 20μl cDNA which is sufficient for the analysis of 4–10 genes by qPCR. To obtain high yields and the best quality of RNA for RNAseq and qPCR, protocols for the extraction of RNA need to be optimised depending on the culture condition and possibly on the transportation or storage method.

Quantitative PCR data need to be normalised using reference genes before accurate gene expression analysis can be performed. The candidate reference gene (RG) expression should not vary between samples under investigation or in response to experimental treatment [9]. Two of the most frequently reported RGs for gene expression data normalisation are beta-actin (*ACTB*) and glyceraldehyde-3-phosphate dehydrogenase (*GAPDH*). Recently, reports advised to forgo these genes identifying them as unsuitable for accurate gene expression normalisation [10, 11], possibly due to the inability to design gene specific primers allowing to discriminate between the actual gene and their numerous (>60) pseudogenes [12]. Several other genes have been reported as normalisers in MSC gene expression analysis. Unfortunately, neither does a universal RG exist nor is there a fixed number of defined RGs to use and these should be determined experimentally [13]. In this report we aimed to identify suitable RGs for gene expression normalisation regardless of cell culture dimension, isolation, storage, or RNA extraction method. RNA has been extracted from two human MSC lines cultured in 2D and 3D by two different methods (RNeasy and Trizol) after storage in RLT-buffer, Trizol or RNAlater and evaluated for quality (gel electrophoresis) and yield (Nanodrop). Thirteen primer sets have been designed and evaluated as candidate RGs using four different ranking approaches. Most rankings indicated *TBP* amongst the most suitable of all candidate RGs for the normalisation of gene expression data from heterogeneous hMSC sample sets. Nevertheless, none of the rankings were the same nor could one single gene be nominated as suitable for normalisation of all comparisons. Therefore, as advocated already by others [9, 14], we strongly recommend to use multiple candidate RGs and all or a selection of the validated primer sets described in this report could be suitable for future quantitative PCR studies involving human MSCs cultured in 2D and/or 3D.

## Materials and methods

All reagents were supplied by ThermoFisher Scientific (Hemel Hempstead, UK) unless otherwise specified.

### Cell culture

All cell culture incubations were done in a humidified 5% CO2 incubator at 37°C.

#### Human bone marrow derived MSCs with enforced TERT expression

MSC-hTERT cells [15] were seeded at 0.5*10^6^ cells/well in DMEM + 10% FBS + 1% p/s (complete DMEM) in a 48-well culture dish or a 12-well culture dish. The fibrinogen-alginate and fibrinogen scaffolds were placed in a 48-well culture dish whereas the polycaprolactone-poly[N-isopropylacrylamide](PCL-PNIPAAM) beads were placed in a 12-well culture dish covering the entire bottom of the well before 0.5*10^6^ cells were applied to the scaffolds and allowed to seed for 3h in the incubator. All wells were incubated for 48h in complete DMEM before performing Alamar Blue staining. After replacing the Alamar Blue staining solution, cells were cultured for another 16h in complete DMEM before adding Trizol and subsequent RNA extraction.

#### Human umbilical cord derived MSCs

This study was approved by the National Research Ethics Service South Central–Oxford C Research Ethics Committee (REC reference number 09/H0606/5+5) and Oxford Radcliffe Biobank (reference number 16/A052). Written informed consent was obtained from all of the participants. Human umbilical cord was obtained from full-term (>36 weeks) caesarean section deliveries or vaginal birth with informed consent of the mother in the John Radcliffe Hospital. A portion of umbilical cord (length >20 cm) was placed into a sterile container (250 ml pots, Sterilin UK) with 100ml tissue collecting solution (PBS + 1% p/s). The collection container was kept at 4°C for storage and brought to the laboratory for processing within 24 hours. The umbilical cord segments were dissected after washing twice in PBS. Wharton's Jelly tissue of umbilical cord was carefully separated from cord lining, vein and arteries first, and subsequently minced into 1 mm^3^ fragments. The minced fragments were placed into a tissue culture dish and left for attaching without adding culture media for 30 minutes in the incubator. UC-MSC culture media composed of DMEM, 10% foetal bovine serum (FBS), 1% Glutamine and 2ng/ml basic fibroblast growth factor (bFGF; Peprotech, London, UK) was gently added to the culture dish. Media was changed once a week until cells migrating out from the fragments was detected. When outgrown cells reached 70–90% confluency, they were harvested by Trypsin dissociation for further culture or flow cytometry. After culture for 4 days in UC-MSC medium, cells were transferred to three 12-well culture plates (1ml per well) at a concentration of 0.1*10^6^ cells/ml. After an additional culture for 3 days in fresh medium the cells were washed with PBS. Culture plates 2 and 3 were treated with 200μl Trypsin per well for 3 min at 37°C before adding 800μl PBS. The total volume was transferred to tubes and centrifuged for 5 min at 13,000 rpm and subsequently 1ml of either Trizol, RLT-buffer (Qiagen, Manchester, UK) or RNAlater was added to the pallet. RNAlater samples were stored for 24h at 4°C before transferring to either -20°C or -80°C. Cells from plate 1 were treated with 1ml of either Trizol or RLT-buffer (Qiagen). One sample each was used for immediate RNA extraction whereas the other samples were stored at either -20°C or -80°C until RNA extraction.

### Phenotype characterisation of UC-MSCs by flow cytometry

Fluorescent marker conjugated antibodies CD105, CD90, CD73, CD34, and CD31 (R&D Systems, Minneapolis, MN, USA) were used to confirm the phenotypic characteristics and to verify the lack of contaminating cells, according to the markers proposed by the International Society for Cellular Therapy [4]. Non-specific background signal was measured using an isotype control cocktail consisting of mouse anti-human IgG2A, IgG2B and IgG1 (R&D Systems).

Cells were harvested and suspended in flow cytometry buffer (PBS containing 0.5% (w:v) BSA) at a concentration of 4 × 10^6^ cells/ml. Every 2x10^5^ cells were incubated with 10μl of fluorescent marker conjugated antibody at 4°C for 30 min in the dark. The cells were then washed and finally resuspended in 400μl of flow cytometry buffer for analysis. To determine the viability, 1μl Propidium iodide (PI; Sigma-Aldrich, Dorset, UK) was added to each tube just before the sample was analysed using BD FACS Canto-F60 cell sorter (BD Biosciences, Wokingham, UK). Data was analysed by Flowjo software (FlowJo LLC, Ashland, OR, USA).

### Alamar blue staining

Fibrinogen-alginate and fibrinogen scaffolds were transferred to a new well. Cells cultured in 2D and on beads were washed with PBS once. Alamar Blue stain (10% v/v) was added to the wells and incubated for 3h at 37°C before absorbance was measured at 570nm on a SpectraMax i3x Multi-Mode Microplate Reader (Molecular Devices, San Jose, CA, USA). Blank wells without cells were used as negative controls.

### RNA extraction

Samples stored in RNAlater were centrifuged and the supernatant removed before adding either Trizol or RLT-buffer (Qiagen). Cell lysates in Trizol were subjected to RNA extraction as described previously [16]. RNA from samples in RLT-buffer were extracted using the RNeasy kit per manufacturer’s protocol. RNA quantity was determined using a Nanodrop One spectrophotometer. RNA quality was assessed by gel electrophoresis using approximately 0.5μg RNA per sample in a 1% agarose in 1% TAE buffer gel containing 3.5μl SybrSafe per 100 ml gel.

### cDNA generation and standard curve composition

The QuantiTect Reverse Transcription kit (Qiagen) was used to convert 1–4 μg RNA into cDNA per manufacturer’s instructions. Reaction volumes were supplemented with RNAse/DNAse-free water to a final volume of 100μl. For the standard curve to evaluate PCR efficiency, S1 was generated with a pool of 5μg RNA converted into cDNA from all PCL-PNIPAAM beads and 2D 12-wells samples (n = 6) resulting in a final volume of 600μl. Subsequently a 4-fold dilution series was prepared starting with 150μl S1 in 450μl RNAse/DNAse-free water until S5. A no template control (NTC) consisted only of RNAse/DNAse-free water. All cDNA samples were stored at -20°C until processed by qPCR. cDNA was used for qPCR without concentration measurements.

### Quantitative PCR

Primers were designed (Primer-BLAST) if not available from previous papers or not within set ranges and compliant with in silico testing. All primers have been assessed for specificity (BLAST), dimers and hairpins (OligoAnalyzer 3.1; Tm of hairpin <60C, dimers ΔG5’ < 5 kcal/mol; ΔG3’ < 3 kcal/mol) and splice variant detection if applicable (Primer-BLAST). All amplicons have been screened for secondary structures [mFOLD;[17]]. Full details according to MIQE guidelines [13] can be found in Table 1.

**Table 1:** Primer properties and in silico evaluation.

Gene ^a^ (Gene ID)	Gene full name	Acc #	isoforms^b^				PGs^c^ (#)	Primer sequence	Exon	Location	amplicon size (bp)	mFOLD at Ta ^d^ (kcal/mol)
			All	D	PC	PCD						
ACTB (ID: 60)	actin beta	NM_001101	19	9	11	5	18	F 5'-CACCAACTGGGACGACAT-3'	3	420–437	189	-1.43
								R 5'-ACAGCCTGGATAGCAACG-3'	4	591–608		
B2M (ID: 567)	beta-2-microglobulin	NM_004048	14	7	3	3	0	F 5'-TAGCTGTGCTCGCGCT-3'	1	50–65	224	-0.22
								R 5'-AGACCAGTCCTTGCTGAAAGA-3'	2	253–273		
GAPDH (ID: 2597)	glyceraldehyde-3-phosphate dehydrogenase	NM_002046	11	10	6	5	64	F 5'-GGATTTGGTCGTATTGGG-3'	3	104–121	205	0.13
								R 5'-GGAAGATGGTGATGGGATT-3'	4	290–308		
GUSB (ID: 2990)	glucuronidase beta	NM_000181	14	5	2	2	18	F 5'-GGGCCGTTGTTGTGGG-3'	1	168–183	218	-3.60
								R 5'-TCATTGAAGCTGGAGGGAAC-3'	2	366–385		
HMBS (ID: 3145)	hydroxymethylbilane synthase	NM_000190	26	16	11	8	0	F 5'-CCATGTCTGGTAACGGCA-3'	1	156–173	142	0.47
								R 5'-GGGTACGAGGCTTTCAATGT-3'	3	278–297		
HPRT1^(15)^ (ID: 3251)	hypoxanthine phosphoribosyltransferase 1	NM_000194	3	3	1	1	3	F 5'-GACCAGTCAACAGGGGACAT-3'	4	489–508	132	0.73
								R 5'-CCTGACCAAGGAAAGCAAAG-3'	6	601–620		
PPIA^(11)^ (ID: 5478)	peptidylprolyl isomerase A	NM_021130	10	6	5	4	79	F 5'-GTCAACCCCACCGTGTTCTT-3'	1	93–112	97	-1.20
								R 5'-CTGCTGTCTTTGGGACCTTGT-3'	2	169–189		
PUM1 (ID: 9698)	pumilio RNA binding family member 1	NM_001020658	23	9	13	8	0	F 5'-CAGGACATTCACAGACACCA-3'	14	2371–2390	196	-2.19
								R 5'-CGCAAACGAGAGGAAGAGA-3'	15	2548–2566		
RPL13A (ID: 23521)	ribosomal protein L13a	NM_012423	14	9	3	2	25	F 5'-GGATAAGAAACCCTGCGACAA-3'	1	25–45	187	-2.46
								R 5'-GCCAGAAATGTTGATGCCTTC-3'	3	191–211		
RPLP0^(11)^ (ID: 6175)	ribosomal protein lateral stalk subunit P0	NM_053275	27	10	12	5	12	F 5'-CAGCAGGTGTTCGACAATGG-3'	6	805–824	214	-0.91
								R 5'-GTGGGAAGGTGTAATCCGTCT-3'	7	998–1018		
TBP (ID: 6908)	TATA-box binding protein	NM_003194	8	6	7	5	0	F 5'-ATCAGAACAACAGCCTGCC-3'	2	284–302	113	-0.13
								R 5'-GGTCAGTCCAGTGCCATAAG-3'	3	377–396		
TFRC (ID: 7037)	transferrin receptor	NM_003234	15	2	4	2	0	F 5'-CTGGCTCGGCAAGTAGATG-3'	3	356–374	234	0.00
								R 5'-TGCCAGTCTCTCACACTCA-3'	4	571–589		
YWHAZ (ID: 7534)	tyrosine 3-monooxygenase/tryptophan 5-monooxygenase activation protein zeta	NM_145690	23	14	18	11	11	F 5'-TCATCTTGGAGGGTCGTCT-3'	2	381–399	180	-0.04
								R 5'-GACTTTGCTCTCTGCTTGTG-3'	3	541–560		
ENG (ID: 2022)	endoglin	NM_001114753	5	2	3	2	0	F 5'-CCCAAAACCGGCACCCTCA-3'	12		238	
								R 5'-TGGGGGAACGCGTGTGC-3'	14/15			
NT5E (ID: 4907)	5'-nucleotidase ecto	NM_002526	5	1	5	1	0	F 5'-GGCTGCTGTATTGCCCTTTG-3'	7		175	
								R 5'-TACTCTGTCTCCAGGTTTTCGG-3'	8			
THY1 (ID: 7070)	Thy-1 cell surface antigen	NM_006288	10	8	5	3	0	F 5'-AGCATCGCTCTCCTGCTAAC-3'	2		230	
								R 5'-CTGGTGAAGTTGGTTCGGGA-3'	3			

Details of primers used in this manuscript.

^a^Previously published primer sets are referenced. All others have been newly designed.

^b^According to Ensemble.com (D = detectable with designed primers; PC = Protein Coding; PCD = Detectable PC with designed primers)

^c^PGs = Known pseudogenes. According to NCBI Gene database

^d^In case of multiple possible structures the most negative value is given; **Bold** = in primer annealing region 3’/5’.

Quantitative PCR was based on intercalating dye technology using SyGreen and a passive reference ROX (PCRBiosystems, London, UK) on an Applied Biosystems StepOnePlus Real-Time PCR system according to the manufacturer’s instructions in 96-well plates and plate seals. Primers (Sigma-Aldrich) had a final concentration of 400nM each. Standards, NTCs and samples were all done in duplicate. For each reaction, 2μl cDNA template was used in a 20μl final reaction volume containing 3mM Mg^2+^ and no Na^+^. Reactions started with 3 min at 95°C, followed by 40 cycles of 15 s at 95°C and 30 s at Tm. This reaction was followed by a melting curve, stepwise increasing temperature each 15 s by 0.5°C, ranging from 65°C to 95°C. Optimal Tm was determined using a temperature gradient ranging from 56°C to 66°C on a 4-fold dilution series using cDNA derived from all PCL-PNIPAAM beads and 2D 12-wells samples. Baseline was set automatically and well specific during analysis. All samples for a particular gene were in the same plate and all plates contained the standard curve.

### Statistical analysis

All statistical analyses were performed using GraphPad Prism 8. Raw Cq values obtained from StepOne software were used for validating the primer sets and ranking their stability using the web-based free analysis program RefFinder [18] integrating BestKeeper [19], the delta Ct method [20], GeNorm [9], and NormFinder [21]. Cited references describe the statistics and manipulations in detail. The ΔΔCq values for the target genes (i.e. *NT5E*, *THY1*, *ENG*) were normalised against different reference genes (or a combination of reference genes). Only samples with valid ΔΔCq levels across all studied sets were used for grouped correlation analysis in MATLAB. The reference gene sets that were used to normalise the target genes were either *TBP*, *YWHAZ*, and *RPL13A* (most stably expressed), *PPIA*, *HPRT1*, and *B2M* (least stably expressed), the two most stably expressed genes (*TBP* and *YWHAZ*), or a single reference gene (*TBP* or *YWHAZ*). The Pearson coefficient (R) of the target genes ΔΔCq values was calculated for each sample against the remaining sample set. The R for each sample against the samples from different groups (i.e., 2D UC-MSCs, 2D MSC-hTERT, or 3D MSC-hTERT) were averaged as the relative correlation against that particular group of samples and subsequently referenced as the grouped-correlation coefficient. The grouped-correlation coefficient for each sample was plotted on a 3-dimensional scatter plot.

## Results and discussion

**Fig 1:**
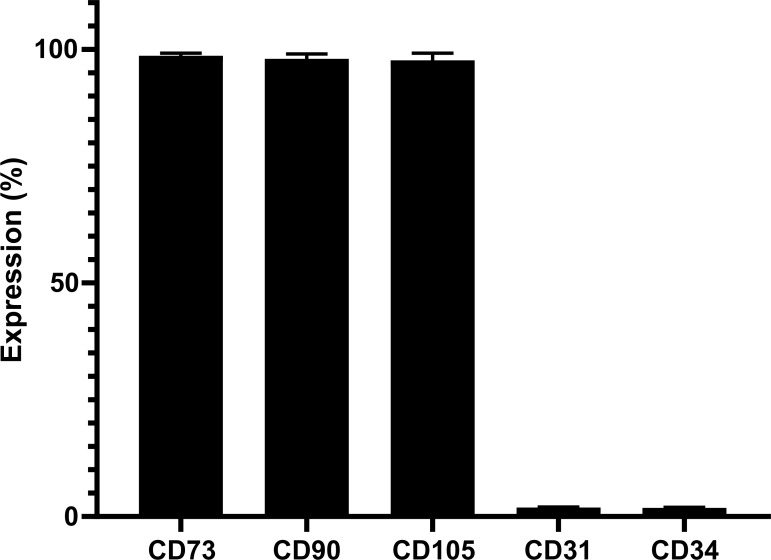
Flow cytometry analyses of UCMSC surface markers. Fluorescently-labelled antibodies were used to detect CD73, CD90, CD105, CD31, and CD34 expression levels by flow cytometry. The results indicated the genuine MSC character expected for cells derived from umbilical cord (UC). Error bars indicate standard deviation.

MSCs, immortalized by transduction with human telomerase reverse transcriptase (hTERT), referred to as MSC-hTERT [15] and MSCs derived from umbilical cord (UC) were cultured to obtain a variety of RNA samples. To generate these UC derived MSCs (UC-MSCs), fresh umbilical cord has been obtained and cells were able to adhere to plastic culture flasks. Flow cytometry indicates high levels of typical MSC markers CD73, CD90, CD105 and very low levels of CD31 and CD34 (Fig 1) indicating a genuine MSC phenotype [4].

**Fig 2:**
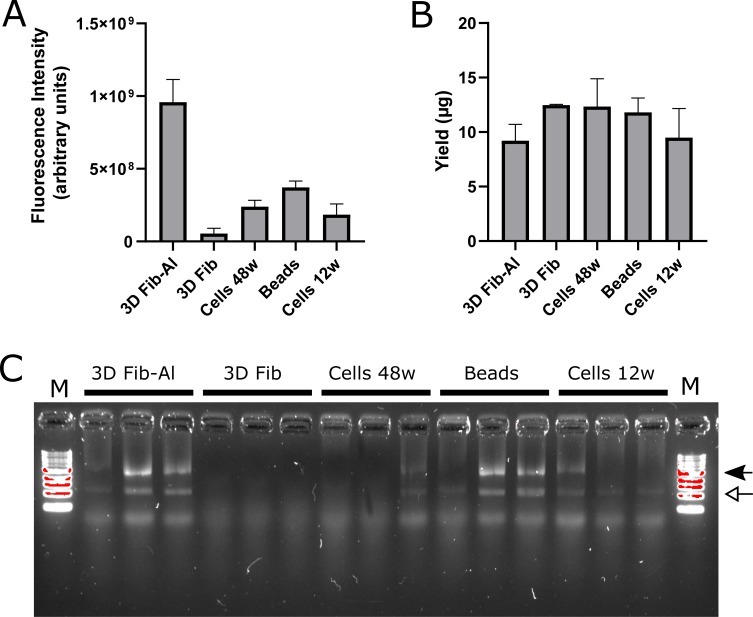
Cells cultured in 3D scaffolds and 2D monolayer culture dish. Cells cultured in fibrinogen-alginate (3D Fib-Al) and fibrinogen (3D Fib) scaffolds, on PCL-PNIPAAM (Beads) or in 48 well (Cells 48w) and 12 well (Cells 12w) culture plates (All n = 3) were stained for their viability with Alamar Blue (A). Arbitrary units are given on the y-axis. RNA concentration (B) from Trizol extraction for the same samples is given (μg) on the y-axis. Error bars represent the standard deviation. Gel electrophoresis (C) indicated separation of the 28S and 18S RNA units in most samples (locations indicated by closed or open arrow, resp.). Sample lanes are flanked by a 1kb DNA ladder (M).

RNA samples from MSC-hTERT and UC-MSC cultures were obtained using different extraction protocols (RNeasy and Trizol) after cell storage in different media (RLT, Trizol, and RNAlater) for several time periods (0 days, 7 days, and 30 days) to identify candidate RGs for accurate normalisation of gene expression in human MSCs cultured in 2D and 3D (Table 2 and Fig 2A). This variability in cell isolation, storage and RNA extraction method resulted in very similar RNA yields and quality (Table 2). One sample (Table 2, III.B2) indicated no or very little RNA and was excluded for further analysis. Although the A260/A280 ratios for the MSC-hTERT samples were somewhat lower, RNA yield was proportional to the starting cell amount when compared with the UC-MSCs.

**Table 2:** Sample diversity.

	Sample Name	Cell # at start culture	Culture time (days)	Cell isolation	Storage compound	Storage time (days)	Storage temp. (°C)	Extraction method	RNA total (μg)	A260/A280	A260/A230
1	3D Fib-Alg 1	5.00E+05	3	Trizol	Trizol	0	N/A	Trizol	7.51	1.477	0.389
2	3D Fib-Alg 2	5.00E+05	3	Trizol	Trizol	0	N/A	Trizol	9.67	1.487	0.448
3	3D Fib-Alg 3	5.00E+05	3	Trizol	Trizol	0	N/A	Trizol	10.42	1.495	0.424
4	3D Fib 1	5.00E+05	3	Trizol	Trizol	0	N/A	Trizol	12.43	1.519	0.380
5	3D Fib 2	5.00E+05	3	Trizol	Trizol	0	N/A	Trizol	12.42	1.524	0.406
6	3D Fib 3	5.00E+05	3	Trizol	Trizol	0	N/A	Trizol	12.55	1.553	0.394
7	Cells 48w 1	5.00E+05	3	Trizol	Trizol	0	N/A	Trizol	12.27	1.520	0.412
8	Cells 48w 2	5.00E+05	3	Trizol	Trizol	0	N/A	Trizol	14.92	1.536	0.432
9	Cells 48w 3	5.00E+05	3	Trizol	Trizol	0	N/A	Trizol	9.84	1.467	0.403
10	Beads 1	5.00E+05	3	Trizol	Trizol	0	N/A	Trizol	12.97	1.507	0.442
11	Beads 2	5.00E+05	3	Trizol	Trizol	0	N/A	Trizol	12.07	1.513	0.418
12	Beads 3	5.00E+05	3	Trizol	Trizol	0	N/A	Trizol	10.39	1.484	0.411
13	Cells 12w 1	5.00E+05	3	Trizol	Trizol	0	N/A	Trizol	7.50	1.490	0.504
14	Cells 12w 2	5.00E+05	3	Trizol	Trizol	0	N/A	Trizol	8.47	1.461	0.392
15	Cells 12w 3	5.00E+05	3	Trizol	Trizol	0	N/A	Trizol	12.52	1.541	0.433
16	I.A3	1.00E+05	3	RLT	RLT	0	N/A	Rneasy	2.16	2.018	1.550
17	I.B4	1.00E+05	3	RLT	RLT	7	-80	Rneasy	2.52	2.047	1.563
18	I.B3	1.00E+05	3	RLT	RLT	7	-20	Rneasy	2.46	2.053	1.237
19	I.C4	1.00E+05	3	RLT	RLT	30	-80	Rneasy	2.13	2.272	0.061
20	I.C3	1.00E+05	3	RLT	RLT	30	-20	Rneasy	2.83	2.095	0.421
21	I.A2	1.00E+05	3	Trizol	Trizol	0	N/A	Trizol	1.93	2.125	1.088
22	I.B2	1.00E+05	3	Trizol	Trizol	7	-80	Trizol	2.65	2.056	1.931
23	I.B1	1.00E+05	3	Trizol	Trizol	7	-20	Trizol	1.16	2.054	1.362
24	I.C2	1.00E+05	3	Trizol	Trizol	30	-80	Trizol	0.92	2.035	0.669
25	I.C1	1.00E+05	3	Trizol	Trizol	30	-20	Trizol	3.28	2.046	1.564
26	II.A3	1.00E+05	3	Trypsin	RLT	0	N/A	Rneasy	2.39	2.046	1.730
27	II.B4	1.00E+05	3	Trypsin	RLT	7	-80	Rneasy	2.73	2.105	1.345
28	II.B3	1.00E+05	3	Trypsin	RLT	7	-20	Rneasy	3.51	2.081	1.804
29	II.C4	1.00E+05	3	Trypsin	RLT	30	-80	Rneasy	1.69	2.156	0.679
30	II.C3	1.00E+05	3	Trypsin	RLT	30	-20	Rneasy	2.59	2.092	1.031
31	III.B4	1.00E+05	3	Trypsin	RNAlater	7	-80	Rneasy	1.47	2.160	0.991
32	III.B3	1.00E+05	3	Trypsin	RNAlater	7	-20	Rneasy	1.99	2.059	1.261
33	III.C4	1.00E+05	3	Trypsin	RNAlater	30	-80	Rneasy	1.24	2.134	0.597
34	III.C3	1.00E+05	3	Trypsin	RNAlater	30	-20	Rneasy	1.42	2.126	0.587
35	III.B2	1.00E+05	3	Trypsin	RNAlater	7	-80	Trizol	0.02	27.025	0.034
36	III.B1	1.00E+05	3	Trypsin	RNAlater	7	-20	Trizol	1.80	2.071	1.517
37	III.C2	1.00E+05	3	Trypsin	RNAlater	30	-80	Trizol	1.21	1.969	0.990
38	III.C1	1.00E+05	3	Trypsin	RNAlater	30	-20	Trizol	2.54	2.041	1.624
39	II.A2	1.00E+05	3	Trypsin	Trizol	0	N/A	Trizol	2.88	2.021	1.540
40	II.B2	1.00E+05	3	Trypsin	Trizol	7	-80	Trizol	2.93	2.069	1.861
41	II.B1	1.00E+05	3	Trypsin	Trizol	7	-20	Trizol	1.68	2.026	1.449
42	II.C2	1.00E+05	3	Trypsin	Trizol	30	-80	Trizol	1.42	2.045	1.706
43	II.C1	1.00E+05	3	Trypsin	Trizol	30	-20	Trizol	3.10	2.044	2.017

Samples were obtained from MSC-hTERT (1–15) and UC-MSC (16–43) cultured at different cell densities, stored at different temperatures and time, and using different extraction methods. Yield, A260/280, and A260/A230 ratios are given. N/A; Not Applicable.

Viability in cell culture can be assessed using Alamar Blue. The viability of MSC-hTERT samples cultured in 2D in 12-well dishes was comparable to cells cultured in 48-well dishes (Fig 2A). Whereas both types of fibrin scaffolds were transferred to a new plate before Alamar Blue assay, the PCL-PNIPAAM beads were not. Therefore the Alamar Blue level and the RNA yield (Fig 2A) might also have come from cells not attached to the beads but to the surface of the culture dish. The fibrin and fibrin-alginate scaffolds are porous and Trizol might not be able to lyse all cells or be aspirated as much as from the beads or the 2D culture dishes which might explain a reduced RNA yield from the fibrin-alginate scaffold when compared to the beads and 2D cultured cells. Although the fibrin scaffold showed low levels of Alamar Blue staining the RNA yield seemed relatively higher than from the others. Unfortunately the electrophoresis indicated lower amounts of RNA compared to the other samples (Fig 2B) and indeed the Nanodrop readings might have been skewed possibly because of contaminants such as phenol.

The remaining samples (N = 42) were all very different in cell type, culture dimension, storage and extraction method. Because of this variable sample composition 13 genes (*ACTB*, *B2M*, *GAPDH*, *GUSB*, *HMBS*, *HPRT1*, *PPIA*, *PUM1*, *RPL13A*, *RPLP0*, *TBP*, *TFRC*, and *YWHAZ*) were selected to develop primers and evaluate their suitability as reference genes for the accurate normalisation of gene expression data regardless of prior experimental differences. These genes were selected because of their use in many other reports. However, to make sure that the primers met the in silico design requirements (e.g. amplicon size 70 – 250bp, secondary structure energy >-3.0 for primer annealing sites on amplicon, primers separated by at least one intron, etc.) we designed those for which we could not identify previously reported appropriate primer sets (Table 1). Primers were validated by qPCR for their efficiency and optimal annealing temperature (Tm) using a standard curve consisting of a 4-fold dilution series composed of cDNA converted from RNA extracted from cells grown on 12-well culture dishes and beads. From the standard curve plot the slope can be determined and efficiency (Eff.) was calculated according to the equation;

$$Eff.={(10}^{\left(\frac{-1}{slope}\right)}-1)*100\%$$

**Fig 3:**
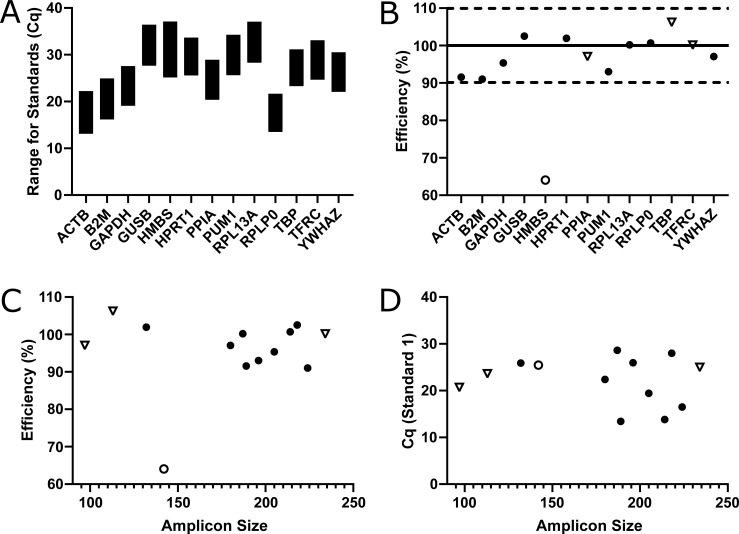
Primer efficiency and gene quantity. All genes could be detected by their respective primer sets (A) of which most were within the set efficiency range of 90–110% (B). Dotted lines define efficiency boundaries and the solid line represents 100% efficiency. Only *HMBS* had a too low efficiency (< 90% which is represented as an open dot). The length of the formed product (amplicon) does not have linear correlation to the efficiency (C; r^2^ = 0.007705, p = 0.7755) or to the abundancy indicated by the Cq of the first standard (D; r^2^ = 0.03130, p = 0.5631). Primer sets potentially having annealing difficulties caused by possible folding of the amplicon as indicated by mFold analysis (Table 1) are depicted as open triangles.

**Fig 4:**
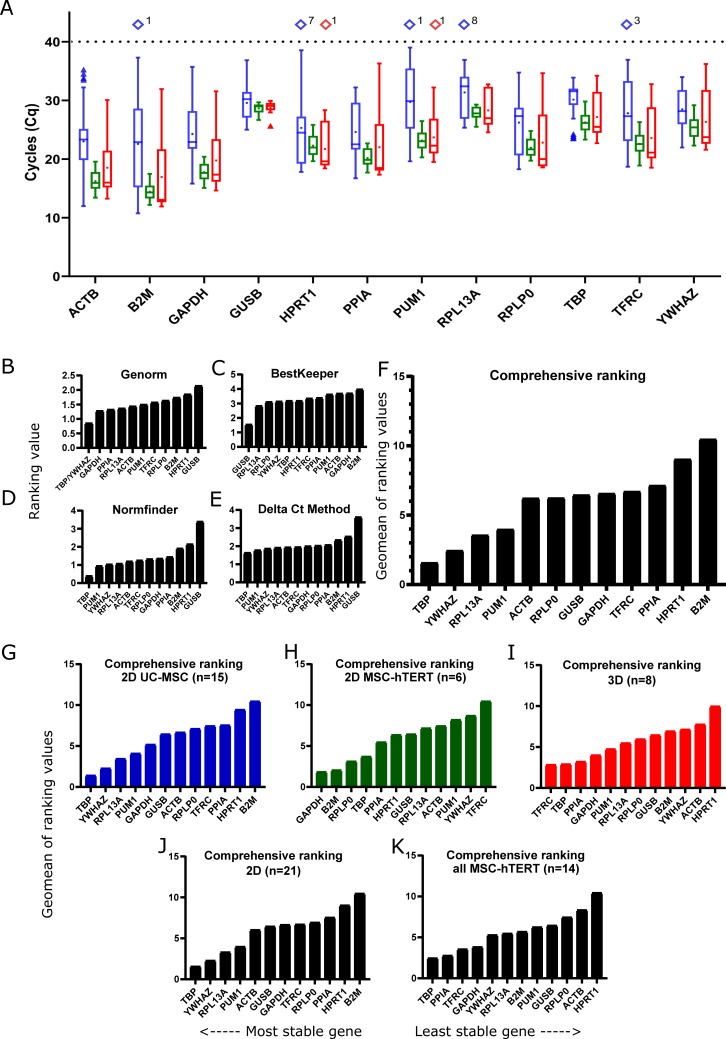
Gene expression and stability. Gene expression detection range (Cq) was analyzed (A) in 2D UC-MSC (blue; n = 27), 2D cultured MSC-hTERT (green; n = 6) and 3D cultured MSC-hTERT (red; n = 9) samples. Samples with no Cq-values (number states amount) are shown as Cq > 40 (diamonds). Black, dotted line is maximum cycles in qPCR run. Boxes represent interquartile range (IQR = Q1 –Q3). Single, triangles represent outliers (Q3+1.5xIQR or Q1-1.5xIQR) if present. Whiskers represent maximum and minimum value after omitting outliers. Means are depicted by dots and medians by horizontal lines in the boxes. Gene expression stability according to Genorm (B), BestKeeper (C), Normfinder (D), and the Delta Ct method (E) were determined as well as the overall comprehensive ranking (F). Comprehensive rankings for all UC-MSC samples (G), only the 2D cultured MSC-hTERT (H), only 3D cultured hMSC-hTERT samples (I), all 2D cultured samples (J), and all hMSC-hTERT samples (K) were calculated using RefFinder.

Some genes were more abundantly expressed than others (Fig 3A) though there was no correlation between amplicon size and efficiency (Fig 3C; r^2^ = 0.007705, p = 0.7755) or quantification cycle (Cq, a.k.a. Ct or Cp) (Fig 3D; r^2^ = 0.03130, p = 0.5631). Also the primer sets with potential annealing difficulties because of possible folding of the amplicon at the annealing site performed equally well as the others (Table 1 and Fig 3B, 3C and 3D). Despite careful design, some primer sets were able to form primer-dimers causing an amplification signal in the NTCs. No primer-dimers were detected in the samples. Nevertheless, the presence of primer-dimers always needs to be evaluated when performing qPCR since they could have had an effect on the overall amplification signal. All primer sets but *HMBS* showed efficiencies between 91–107% (Table 3 and Fig 3) and the remaining primer sets were used to evaluate expression levels in the available samples to determine their stability. Several samples (n = 13) showed undetectable levels of gene expression when analyzing *B2M*, *HPRT1*, *PUM1*, *RPL13A*, and *TFRC* and were excluded for further analysis (Fig 4A). The remaining samples (n = 29) were used to rank these genes (n = 12) according to their stability using the currently available major computational programs GeNorm [[9]; Fig 4B], BestKeeper [[19]; Fig 4C], NormFinder [[21]; Fig 4D] and the delta Ct method [[20]; Fig 4E] integrated in RefFinder [18] resulting in an overall comprehensive ranking (Fig 4F).

**Table 3:** Primer set validation.

Gene ^a^	Optimal reaction Ta (°C)	amplicon Tm (°C)	Eff (%)	r^2^	Cq St1	Cq St5	NTC (Cq)
ACTB	60	88	91.56	0.9889	13.43	22.23	31.49
B2M	60	83	91.04	0.9982	16.49	24.93	37.08
GAPDH	64	84	95.35	0.9942	19.43	27.56	ND
GUSB	62	92	102.53	0.9652	27.98	36.42	37.09
HMBS	62	86.5	64.08	0.9643	25.44	37.08	ND
HPRT1^(15)^	66	80	101.95	0.9965	25.88	33.65	ND
PPIA^(11)^	62	86.5	97.07	0.9993	20.69	28.93	ND
PUM1	66	87	93.02	0.9957	25.94	34.25	ND
RPL13A	66	89.5	100.18	0.9696	28.6	37.04	ND
RPLP0^(11)^	62	85	100.69	0.9980	13.82	21.65	37.15
TBP	64	85	106.21	0.9901	23.62	31.16	33.96
TFRC	62	82.5	100.14	0.9974	24.97	33.08	ND
YWHAZ	64	82	97.05	0.9951	22.66	30.91	ND
ENG	64	88.0	101.87	0.9253	21.85	31.63	ND
NT5E	64	86.0	98.29	0.9921	19.66	27.21	ND
THY1	65	88.0	95.39	0.9966	23.80	31.78	ND

Experimental data per primer set (further in silico information is given in Table 1).

^a^References of previously reported primer sets are given unless newly developed. ND; Not Detected

All but BestKeeper ranked *TBP* as the best reference gene and *YWHAZ* in the top 3. *RPL13A* was found in the top 5 of all rankings whereas *PUM1* was ranked second using Normfinder and the Delta Ct method though amongst the lesser stable genes according to Genorm and BestKeeper. The most commonly used genes for normalisation are *ACTB* and *GAPDH* which are found to be moderately stable except for *GAPDH* ranking third when using Genorm. BestKepper ranked *GUSB* first whereas all the others ranked this gene as the least stable. Also, *HPRT1* was ranked amongst the least stable genes in all but BestKeeper ranking.

As pseudogenes can skew the gene expression of the genuine gene their number in the genome should ideally be absent. The NCBI Gene databank (www.ncbi.nlm.nih.gov/gene) indicates that only *B2M*, *PUM1*, *TBP*, and *TFRC* are devoid of any known pseudogenes whereas the others, and *GAPDH* and *PPIA* in particular, have some or numerous known pseudogenes present in the genome (Table 1). According to Liao and colleagues the actual number of pseudogenes for human *ACTB* is even higher at 64 pseudogenes [12].

As some of the samples were from cultured MSC-hTERT and other samples from hUC-MSC we established a comprehensive ranking for these samples separately. Comparing these rankings (2D MSC-hTERT vs. 2D UC-MSC), striking differences could be observed (Fig 4G and 4H). Not only was *YWHAZ* ranked second in UC-MSC whilst ranked second last for MSC-hTERT, also *B2M* was inversely ranked between the two cell lines in 2D cultures. Furthermore, comparing the 2D cultured MSC-hTERT (Fig 4G) with the 3D cultured MSC-hTERT (Fig 4I), revealed the inverted ranking of *TFRC*; ranked last in 2D MSC-hTERT whilst simultaneously the best reference gene in 3D culture.

When comparing the comprehensive ranking of all samples (Fig 4F) with only 2D (Fig 4J) or only 3D samples (Fig 4I), *TFRC* is ranked in the middle except for the 3D samples where it is considered the most stably expressed gene. Cancer stem cells (CSCs), e.g. from sarcoma, a cancer arising from mesenchymal cells, resemble MSCs in respect to self-renewal potential and differentiation capacity and can be used as a 3D model. *PPIA*, *GAPDH*, and *YWHAZ* have been shown to be amongst the best suitable reference genes for gene expression normalisation in CSC samples [22]. For our 3D cultured MSCs both *PPIA* and *GAPDH* ranked in the top 4 whereas *YWHAZ* ranked amongst the least stable genes. These differences indicate that careful consideration is required when selecting reference genes even when cell types are fairly similar or when culture dimension (e.g. 2D or 3D) is different.

Although in the top 3 for all samples together, *YWHAZ* is an excellent candidate reference gene in hUC-MSC samples, in MSC-hTERT it is amongst the lesser stable genes and for *PPIA* and *GAPDH* the opposite is true. Furthermore, *YWHAZ* ranks in the top 3 for UC-MSCs (Fig 4H) and overall (Fig 4F) whereas it is in the bottom three in both 3D cultured MSC-hTERT (Fig 4I) and 2D-MSC-hTERT (Fig 4G) but back in the top 5 for all MSC-hTERT (Fig 4K) samples. These differences in rankings illustrate the necessity to use multiple candidate reference genes to determine their stability and their usefulness for accurate gene expression normalisation for each and every experiment. As indicated some samples showed no detectable expression levels for some genes (Fig 4A). For the 2D cultured UC-MSCs 1–8 samples (out of 27) no expression was detected in five of the 12 genes and only one sample (out of 9) of the 3D cultured MSC-hTERT showed no detectable expression for two candidate reference genes. The MSC-hTERT cells cultured in 2D (n = 6) showed detectable expression levels for all samples in all genes. Two of these genes (*PUM1* and *RPL13A*) ranked in the overall top five (Fig 4F) and the 2D-UC-MSC (Fig 4H) ranking whereas they dropped to some extent in the other rankings. This further indicates that careful selection and assessment of candidate reference genes is needed depending on cell type.

The literature advises the use of at least three control genes for normalisation [9]. To illustrate the effect of using inappropriate reference genes we performed additional qPCR for MSC marker genes Endoglin (*ENG* or CD105), *THY1* (CD90), and 5'-Nucleotidase Ecto (*NT5E* or CD73) (primer details in Table 1) and implemented normalisation using the three most stably (*TBP*, *YWHAZ*, and *RPL13A*) or least stably (*PPIA*, *HPRT1*, and *B2M*) expressed reference genes. The respective standard deviations increased when the least stably expressed reference genes were used (S1 Fig). The effect was less pronounced or even adverted for the MSC-hTERT samples since the three most stably expressed reference genes for these samples were *TBP*, *PPIA*, and *TFRC*. The top three ranked genes for the UC-MSCs were the same as for the overall comprehensive top three ranked genes.

**Fig 5:**
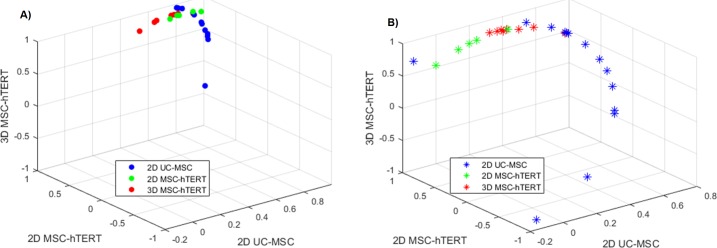
Relative grouped-correlation for each sample against the three different sample groups. The data normalised against the three most stable reference genes is shown in a), represented by solid spheres. The data normalised against the three least stable reference genes is shown in b), represented by asterisks. Colour coding for the three groups: Blue, 2D UC-MSC; green, 2D MSC-hTERT; red, 3D MSC-hTERT.

The three sample groups (2D UC-MSC, 2D MSC-hTERT, and 3D MSC-hTERT) investigated showed much better correlation between the delta-delta Cq (ΔΔCq) values when using the three most stably expressed reference genes compared to the least stably expressed reference genes (Fig 5 and S2 Fig). The coefficient of variance (CoV) was also much lower when using the best reference genes (Table 4). Nevertheless, the CoVs for the three groups further decreased when only *TBP* and *YWHAZ* were used for normalisation and even more so when using only *YWHAZ*. In contrast, using *TBP* alone for normalisation resulted in an increase of the CoV despite this gene being the most stably expressed gene for most of the rankings. As noted earlier, the use of multiple reference genes is advised. Although gene expression normalisation with only *YWHAZ* resulted in the lowest CoV between samples in all groups, we would still recommend the use of multiple reference genes since the results further indicate that the best ranked gene might not be the best single reference gene for normalisation.

**Table 4:** Coefficient of variance for the grouped-correlation coefficients in each of the three sample groups.

	Most Stable	Least Stable	TBP+YWHAZ	single-TBP	single-YWHAZ
2D UC-MSC	0.075	0.623	0.027	0.136	0.014
2D MSC-hTERT	0.013	0.032	0.006	0.012	0.002
3D MSC-hTERT	0.029	0.038	0.012	0.042	0.008

The coefficient of variance data normalised against different sets of reference genes is shown in each column.

Potentially, all candidate reference genes are still suitable for gene expression normalisation. Less stable in this experiment could be the favorite gene for another experiment. All or a selection of the validated primer sets can be used for future experiments to perform accurate gene expression normalisation, although it is advised to always include *TBP* in these assessments.

## Conclusion

To accurately normalise gene expression data a panel of 12 reference genes has successfully been developed and assessed for their stability in hMSC cultured in 2D and 3D. Differences between 2D and 3D cultured samples and the two cell lines used can be observed in the ranking of the candidate reference genes indicating the influence of experimental properties and careful selection of reference genes for normalisation. The inconsistency between rankings further illustrates the necessity of using multiple reference genes for normalisation and moreover, no universally suitable gene for normalisation exists. This panel of validated candidate reference genes is available for gene expression analysis of MSCs although determining which genes to use needs to be established per experiment. Devoid of any known pseudogenes and amongst the most stable genes in all rankings, *TBP* is recommended for inclusion in any reference gene assessment in hMSC culture experiments.

## Supporting information

S1 Fig MSC marker gene expression normalization. MSC markers (*NT5E*, *THY1*, and *ENG*) were analysed and their Cq values normalised with the average of the three most stably expressed RGs (solid bars) or the three least stably expressed RGs (open bars) according to the overall comprehensive ranking. Error bars indicate standard deviation (SD). Gene specific means, SD, and sample numbers (N) are given in the tables below each graph.
(EPS)

S2 Fig The relative correlation of the ΔΔCq values for the three target genes against the samples from the same group. The relative correlation values after the normalization using the three most stably expressed genes or the three least stably expressed genes were grouped separately. A) 2D UC-MSC group. B) 2D MSC-hTERT group. C) 3D MSC-hTERT group.
(EPS)
